# Story of Rubidium-82 and Advantages for Myocardial Perfusion PET Imaging

**DOI:** 10.3389/fmed.2015.00065

**Published:** 2015-09-11

**Authors:** Jean-François Chatal, François Rouzet, Ferid Haddad, Cécile Bourdeau, Cédric Mathieu, Dominique Le Guludec

**Affiliations:** ^1^Groupement d’Intérêt Public Arronax, University of Nantes, Saint-Herblain, France; ^2^UMR 1148, Department of Nuclear Medicine, Bichat Hospital, Assistance Publique Hôpitaux de Paris, DHU FIRE (Fibrosis, Inflammation, Remodeling in Cardiovascular, Respiratory and Renal Diseases), Paris-Diderot University, Paris, France; ^3^Department of Nuclear Medicine, Nantes University Hospital, Nantes, France

**Keywords:** rubidium-82, myocardial perfusion, PET imaging

## Abstract

Rubidium-82 has a long story, starting in 1954. After preclinical studies in dogs showing that myocardial uptake of this radionuclide was directly proportional to myocardial blood flow (MBF), clinical studies were performed in the 80s leading to an approval in the USA in 1989. From that time, thousands of patients have been tested and their results have been reported in three meta-analyses. Pooled patient-based sensitivity and specificity were, respectively, 0.91 and 0.90. By comparison with ^99m^Tc-SPECT, ^82^Rb PET had a much better diagnostic accuracy, especially in obese patients with body mass index ≥30 kg/m^2^ (85 versus 67% with SPECT) and in women with large breasts. A great advantage of ^82^Rb PET is its capacity to accurately quantify MBF. Quite importantly, it has been recently shown that coronary flow reserve is associated with adverse cardiovascular events independently of luminal angiographic severity. Moreover, coronary flow reserve is a functional parameter particularly useful in the estimate of microvascular dysfunction, such as in diabetes mellitus. Due to the very short half-life of rubidium-82, the effective dose calculated for a rest/stress test is roughly equivalent to the annual natural exposure and even less when stress-only is performed with a low activity compatible with a good image quality with the last generation 3D PET scanners. There is still some debate on the relative advantages of ^82^Rb PET with regard to ^99m^Tc-SPECT. For the last 10 years, great technological advances substantially improved performances of SPECT with its accuracy getting closer to this of ^82^Rb/PET. Currently, the main advantages of PET are its capacity to accurately quantify MBF and to deliver a low radiation exposure.

## Introduction

The story of medical use of rubidium goes back in 1954 when Love et al. showed that the biological behavior of rubidium was comparable to the one of potassium and that its myocardial muscle uptake was proportional to blood flow in coronary arteries (1). Following this paper, several preclinical studies have been performed mainly in dogs and using different radionuclides of rubidium until the early 80s when the first injection in humans took place (2). During the 80s, a few clinical studies, including hundreds of patients, demonstrated the good diagnostic accuracy of ^82^Rb/PET, which was higher than the one obtained with ^99m^Tc/SPECT (3, 4). Subsequently, a generator ^82^Sr/^82^Rb (CardioGen-82^®^) was approved in 1989 and delivered in the USA by Bracco Diagnostics, Inc., for clinical use.

At that time, the number of PET cameras, their technological performances, and the production capacities of strontium-82 were limited, explaining a slow progression of this technology in the USA. However, despite this limitation, thousands of patients were tested with PET using rubidium-82 allowing three meta-analyses to be performed.

During the last 10 years, with the increased number of PET/CT systems and the higher strontium-82 production capabilities, the number of patients injected with rubidium-82 in the USA dramatically grew even if it still represents a small percentage by comparison with the use of ^99m^Tc-sestamibi and ^99m^Tc-tetrofosmin. Moreover, for the last 10 years, great technological advances have been introduced, including semiconductor detector SPECT scanners, novel collimator design, and novel iterative reconstruction methods allowing to substantially improve count sensitivity and image resolution. Consequently, the diagnostic accuracy of ^99m^Tc/SPECT got closer to this of ^82^Rb/PET, opening a large debate on the advantages of one technique with regard to the other.

The latest clinical developments zeroed in on the high prognostic value of quantification using PET and allowing to accurately measure myocardial blood flow (MBF) and coronary reserve. Even if there are no real technical limitations to such measurements with SPECT it will take some years before their potential clinical validation (5). Finally, the level of radiation dose is an important parameter to be taken into consideration due to the high number of patients to be tested and the repetition of imaging in the same patients. The aforementioned technological advances allowed to significantly reduce radiation doses with both ^99m^Tc/SPECT and ^82^Rb/PET even if the latter delivers the smallest dose (6).

## Preclinical Studies

Beta- and gamma-emitting rubidium-86 was first used because its long 18.7 days half-life enabled to perform long-lasting kinetic studies. In 1959, Love et al. showed, in 19 dogs, that myocardial uptake of this radionuclide was directly proportional to MBF (7). These results were confirmed 2 years later in 26 dogs by Levy et al. (8).

More than a decade later, Nishiyama et al. compared optimal settings of scintillation camera with ^201^Tl (T1/2:73.1 h, γ: 167 keV), ^43^K (T1/2: 22.2h, γ: 373 keV), ^129^Cs (T1/2: 32.06h, γ:372 keV), and ^81^Rb, a positron-emitting radioisotope with a half-life of 4.6 h (9). Thallium-201 was considered as the best suited with the available equipment at that time and cesium-129 was next best. High-energy photons from ^81^Rb largely made it impossible to obtain an interpretable image without the addition of more shielding.

In 1979, Yano et al. compared several ion-exchange columns to be used in an automated ^82^Sr/^82^Rb generator for testing in man (10).

Finally, in 1982, Selwyn et al. examined the relation between myocardial perfusion and rubidium-82 uptake during acute ischemia in six dogs after coronary stenosis and in five volunteers and five patients with coronary artery disease. Myocardial tomograms, recorded at rest and after exercise in the volunteers showed homogeneous uptake in reproducible and repeatable scans. An absolute mean decrease of 36 ± 14% in regional myocardial uptake was found after exercise in the patients with coronary artery disease (2).

## Clinical Studies

### Meta-analyses of PET studies

Since the approval by FDA in the USA in 1989, a vast amount of clinical studies have been performed, including thousands of patients. Results have been analyzed in three meta-analyses published in 2008 and 2012 (Table 1). Pooled patient-based sensitivity and specificity were, respectively, 0.91 and 0.90. These excellent results should be tempered by some limitations and biases inherent to meta-analyses. There was heterogeneity between studies in scanning protocols and prevalence stenosis with invasive coronarography. Moreover, baseline characteristics, such as gender or disease prevalence, were different between patient populations resulting in a cautious interpretation.

**Table 1:** **Results of meta-analyses with ^82^Rb PET**.

Number of studies/patients	Patient-based sensitivity	Patient-based specificity	Reference
11/1175	0.93 (0.85–0.96)	0.90 (0.75–1.00)	(11)
11/NS^a^	0.84 (0.81–0.87)	0.81 (0.74–0.87)	(12)
6/843	0.91 (0.86–0.96)	0.93 (0.60–1.00)	(13)
28/2018	0.91	0.90	

*NS, not specified*.

*^a^Pooled results*.

For a long time, PET studies have been performed with two-dimensional cameras needing to inject a relatively high activity of rubidium-82 (40 mCi for stress-only) and resulting in non-negligible radiation exposure. The accuracy, outcomes, and cost-effectiveness of 3D PET technology using a low activity of 20 mCi were recently evaluated in seven centers (14). Through an effective standardization and quality assurance program, the image interpretation was highly repeatable in involved centers.

### Meta-analyses of SPECT studies

A much larger number of 227 studies using ^99m^Tc/SPECT and including more than 14,500 patients have been considered in 7 meta-analyses published between 1998 and 2012 (Table 2). Pooled patient-based sensitivity and specificity were, respectively, 0.88 and 0.67. The same limitations as with PET studies were applied to SPECT studies.

**Table 2:** **Results of meta-analyses with ^99m^Tc SPECT**.

Number of studies/patients	Patient-based sensitivity	Patient-based specificity	Reference
20/3474	0.90 (0.89–0.90)	0.77 (0.72–0.83)	(15)
27/3237	0.87^a^	0.64^a^	(16)
44/2837	0.89 (0.82–0.90)	0.75 (0.65–0.75)	(17)
10/651	0.88^a^	0.67^a^	(18)
13/2922	0.81^a^	0.65^a^	(19)
105/NS	0.88 (0.88–0.89)	0.61 (0.59–0.62)	(12)
8/1410	0.85 (0.72–0.97)	0.82 (0.76–0.92)	(13)
227/14531	0.88 (0.81–0.90)	0.67 (0.61–0.82)	

*NS, not specified*.

*^a^Pooled results*.

### Comparison between PET and SPECT

Myocardial perfusion imaging is a real challenge in overweight or obese patients and in women with large breasts due to attenuation artifacts, resulting in decreased specificity using ^99m^Tc SPECT. PET imaging has improved specificity owing to better spatial resolution, coincidence detection, and accurate attenuation correction.

Bateman et al. compared ^99m^Tc SPECT in 112 patients with ^82^Rb PET in 112 patients (20). They showed a much better diagnostic accuracy using PET, in obese patients with body mass index (BMI) ≥30 kg/m^2^ (85 versus 67% with SPECT). However, it should be noted that SPECT was performed at that time without attenuation correction. With the introduction of iterative reconstruction, SPECT is now performed with attenuation correction, which may correct some artifacts and improve diagnostic accuracy in the obese population but with a decrease of sensitivity. A recent study reported on the value of ^82^Rb PET in 2687 obese patients with BMI ≥30 kg/m^2^ by comparison with 2047 overweight patients (BMI: 25.0–30.0 kg/m^2^) and 1303 normal patients (BMI: <25.0 kg/m^2^). Interestingly, the results showed the same prognostic value irrespective of BMI (21).

In the future, ^82^Rb PET should be compared to contemporary ^99m^Tc SPECT using the last technological advances in the same overweight or obese patients to confirm or not the higher prognostic value of PET to SPECT.

As aforementioned, a large number of clinical studies have been performed using PET and SPECT in different populations of patients allowing to group the results in meta-analyses with the possibility of biases in interpretation of these results.

The most informative comparison between PET and SPECT should be in the same population of patients injected with both radiopharmaceuticals. Such comparisons have been made a long time ago at the time of approval of rubidium-82 in the USA in three studies, including a total of 433 patients (22–24). In fact, these studies used thallium-201 for SPECT, which has been replaced by technetium-99m. Quite recently, a comparison of both modalities has been performed in a small cohort of 27 patients using the most recent hybrid imaging technology, which included CT-based attenuation correction for SPECT and PET (25). ^82^Rb/PET imaging was performed as a second-line test when previous gated rest/stress ^99m^Tc/SPECT with or without attenuation correction was non-conclusive. In this clinical situation, there were much fewer non-conclusive results with PET than with SPECT. Image quality and interpretive confidence were higher with PET than with SPECT even when SPECT was performed with attenuation correction.

### Dosimetric studies

Radiation dosimetry of rubidium-82 has been recently estimated in 10 healthy volunteers using the OLINDA/EXM 1.0 dosimetry software (26). Using different methodological approach, the estimates were discrepant with the results previously reported. The highest absorbed dose was delivered to the kidneys but remaining at a quite acceptable level of 1.3 cGy for 2220 MBq (60 mCi) of injected activity. The effective dose calculated for a rest/stress test with an injected activity of 2 MBq × 1480 MBq (2 mCi × 40 mCi) was 3.7 mSv that is roughly equivalent to the annual natural exposure.

In 2015, Dorbala et al. reported on the way to reduce radiation dose with myocardial SPECT and PET imaging (6). Considering the use of last generation of 3D PET scanners and software allowing to inject half activity of rubidium-82 (2 MBq × 740 MBq or 2 mCi × 20 mCi) for a preserved image quality, the calculated effective dose was 1.26 mSv for rest or stress. In the clinical situation of stress-only 3D PET with MBF estimate, the radiation dose would be at an acceptable level of around 1 mSv. This is half of the radiation dose with ^99m^Tc-sestamibi or tetrofosmin using last generation of scanners.

### Quantification studies

The clinical use of quantitative MBF assessment with ^82^Rb/PET started at the end of the previous decade in the USA. In 2009, El Fakhri et al. demonstrated, in 22 subjects including patients with known coronary artery disease or healthy volunteers, that the measurement of absolute quantitation of MBF was feasible, reproducible, and accurate (27). Two years later, Ziadi et al. prospectively evaluated the prognostic value of coronary flow reserve using ^82^Rb/PET in 704 patients and compared the results with semi-quantitative assessment using summed stress scores (28). They showed that quantitative myocardial flow reserve (MFR) was a good predictor of adverse cardiac events independent of the summed stress scores. The same added prognostic value of blood flow quantitation was confirmed 2 years later by Farhad et al. in 351 patients (29).

Several software packages are available for quantification of MBF. A study (RUBY-10) compared them in 48 patients from 10 centers and showed that, using the most common kinetic model, they may be used interchangeably (30).

The value of quantification of absolute MBF using rubidium-82 has been studied in 140 patients after heart transplant and for whom the prognosis depends on allograft vasculopathy (31). It was clearly shown that mean MFR was a significant predictor of future adverse events.

The great interest of blood flow quantification has been documented in diabetes mellitus (32). Among diabetic patients without coronary artery disease, those with impaired coronary reserve had cardiac event rates comparable to those with prior coronary artery disease, whereas those with preserved coronary reserve had cardiac event rates comparable to those of non-diabetics. Moreover, it has been recently shown that “global coronary flow reserve is associated with adverse cardiovascular events independently of luminal angiographic severity and modifies the effect of early revascularization” (33). The angiographic severity was evaluated using the coronary artery disease prognostic index. This important result documents the complementary but distinct association of functional and anatomic coronary abnormalities. A significant interaction was shown between coronary flow reserve and revascularization strategy, which could have large implications in the future for therapeutic strategy.

One other cardiac PET perfusion tracer, ^13^N-ammonia, has been approved in the USA and is being clinically used at a relatively modest level. Its positron range is favorable resulting in a good image resolution and the myocardial extraction fraction is also favorable but its short physical half-life of 9.96 min requires an onsite cyclotron, which is a great limitation for a routine clinical use. Another PET radiotracer, ^18^F-flurpiridaz is currently undergoing a clinical phase III evaluation and is quite promising after its potential approval. It could then be a real competitor to ^82^Rb. A recent excellent review described in depth the characteristics of all PET perfusion radiotracers (34).

## Advantages

### Daily availability of rubidium-82 in nuclear cardiology departments

Like with all generators, rubidium-82 is daily available in a department of nuclear medicine after elution of the column loaded with strontium-82 (Table 3). Such elution can be repeated every 10 min making possible to inject up to 10–15 patients per day depending on the availability of a dedicated cardiac PET/CT and of patient recruitment rate. A ^82^Sr/^82^Rb generator can currently be used for 28–42 days according to the loaded strontium-82 activity. It can be expected that this use will be extended to 60 days in a near future allowing to increase the number of tested patients with the same generator and consequently to decrease the cost of rubidium-82 examination for each patient.

**Table 3:** **Advantages and disadvantages of the use of ^82^Rb PET myocardial imaging**.

Advantages	Disadvantages
Quantification++	Cost++
Good interpretative confidence	Lack of dedicated cardiac PET cameras
Favorable dosimetry	Limited capacity of strontium production
Good diagnostic accuracy	

### Easy interpretation of images due to high count density

The count density and the uniformity of distribution of rubidium-82 in the myocardium are higher with PET than with SPECT using technetium-99m, even using the last technological advances of both techniques (25). Consequently, the interpretative confidence and interreader agreement are higher with PET leading to a higher accuracy. PET cameras do not need the use of collimators, resulting in higher sensitivity and spatial resolution (35).

### Measurement of myocardial blood flow and coronary reserve

**Figure 1:**
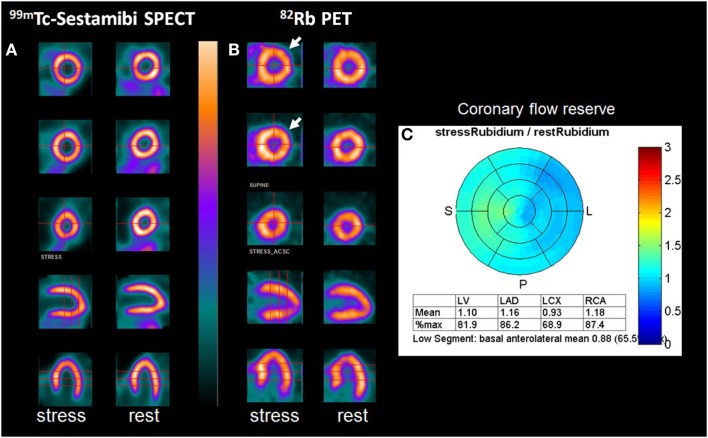
**A 56-year-old woman with a history of obesity (BMI: 31.2 cm/kg^2^), hypertension, hyperlipemia, and type 2 diabetes complicated of retinopathy and renal failure**. The patient was referred to the Nuclear Medicine department for detection of coronary artery disease and underwent both Tc-99m-sestamibi SPECT (D-SPECT, Spectrum Dynamics, Haifa) and rubidium-82 PET/CT (Discovery 690 VCT, GEMS, Buc, France) as part of a clinical trial. SPECT (93% of the predicted maximal heart rate, no symptoms, EKG positive) was normal with a homogeneous uptake of the tracer both at stress and at rest **(A)**. ^82^Rb PET performed after Dipyridamole infusion showed a mild decrease of the tracer uptake in the lateral wall (arrow), completely reversible at rest, raising the suspicion of ischemia in the territory of the circumflex artery **(B)**. This hypothesis was then confirmed by quantitative data derived from myocardial blood flow measurement with a coronary steal phenomenon in the same territory [coronary flow reserve (CFR) <1; **(C)**]. In addition, the CFR was markedly decreased (<1.5) in the territory of both the left descending artery and the right coronary artery, suggesting the presence of significant stenosis of these two coronary arteries. The coronary angiography confirmed the diagnosis of three-vessel disease and the patient underwent surgical revascularization. This case report underlines the greatest sensitivity of ^82^Rb PET over SPECT in the setting of balanced ischemia, in relation with the ability to perform absolute quantification of myocardial blood flow.

Interpretation of SPECT and PET images is visual or semi-quantitative and based on relative uptake. Myocardial areas with the highest uptake are supposed to be supplied by non-obstructive coronary arteries while those with decreased uptake during stress are considered as being supplied by obstructive arteries. A stenosis with a luminal diameter around 50% may be undetected by this visual interpretation. Moreover, patients with subclinical coronary ischemia or microvascular diffuse disease may present only a mild heterogeneous or even homogeneous myocardial uptake. Finally, in the situation of three-vessel coronary disease, a reduction of myocardial uptake may be balanced in all coronary arteries, resulting in a homogeneous left ventricle myocardium at stress (Figure 1).

The measurements of absolute MBF in milliliter per gram per minute and MFR, which is the maximum increase in blood flow above the normal resting volume, allows to complete the field of application of conventional perfusion imaging by identifying subclinical coronary ischemia and characterizing extent and severity in multi-vessel disease. Moreover, it has been clearly shown during the last years that the measurement of hyperemic MBF and MFR using PET/CT may predict, better than other parameters, the occurrence of hard cardiac events.

Today, there is no doubt that absolute myocardial perfusion quantification has been fully validated using PET/CT with ^82^Rb or ^13^N. There is a great debate about the same possibility using ^99m^Tc/SPECT. In a quite recent editorial, Garcia considered the current situation taking into account the most recent technological advances in instrumentation and quantification software (5). New heart-centered SPECT systems have a high count sensitivity and iterative reconstruction enables an efficient correction of attenuation, scatter, and resolution changes with depth. In 2013, Ben-Haim et al. (36) showed the feasibility of measuring MBF and MFR with SPECT. Today, it can be stated that there is no real limitation to the quantification of MBF and MBF reserve with SPECT. The first results on clinical efficacy using conventional SPECT/CT systems have been reported (37) but these preliminary results should be confirmed in large studies and it will take some years before full validation for clinical use. Today, there is no doubt that only PET technology allows to measure rapidly and accurately the MBF.

### Low radiation exposure for patients

Given the high and still increasing number of patients who have myocardial perfusion imaging all over the world each year, the radiation exposure for patients and medical staff is a real concern and should be reduced at the lowest level compatible with a good image quality.

Effective radiation dose is directly related to the physical half-life of the radiopharmaceutical, its biodistribution and injected activity. In this respect, the very short half-life of ^82^Rb of 1.26 min in comparison of 6 h of ^99m^Tc is quite favorable for a low radiation dose, especially if new technologies enable to substantially decrease injected activity. For a long time, using 2D imaging mode, injected activity of rubidium-82 for rest and stress was 1480 MBq (40 mCi), resulting in an estimated dose of 2.5 and 5 mSv, respectively (6). Shifting from 2D to 3D imaging mode allowed to inject half activity (740 MBq or 20 mCi) for the same image quality and then to reduce the dose to 1.3 mSv for stress or rest and even to <1 mSv using new estimates of rubidium-82 dose (26). Such a dose is acceptable when compared to an average natural annual radiation dose of about 3 mSv. Stress-only PET 3D imaging, allowing to measure hyperemic MBF with <1 mSv, would be the preferred strategy for risk stratification if this measurement is clinically validated with regard to the measurement of myocardial blood reserve, which needs both stress and rest tests.

By comparison, effective radiation doses are higher with ^99m^Tc-based radiopharmaceuticals partially owing to the longer half-life of this radionuclide with regard to rubidium-82. Using the most recent advances in cardiac SPECT technology allowing to inject half activity for the same image quality [148–444 MBq (4–12 mCi)], the estimated doses are 2.3 and 8 mSv for rest/stress with ^99m^Tc sestamibi and 2.0 and 6.1 mSv with ^99m^Tc-tetrofosmin (6). Stress-only SPECT 3D imaging would deliver twice higher effective dose than with stress-only PET 3D imaging even if such dose remains moderate.

A recent study reported on projected population cancer risks in the USA using myocardial perfusion scintigraphy (38). The authors considered estimated effective doses ranging from 9 mSv for stress-only technetium-99m to 35 mSv for a rest/stress dual-isotope study associating thallium-201 and technetium-99m and using conventional SPECT equipment. For rubidium-82 PET imaging, they considered an estimated effective dose of 15 mSv for a rest/stress test using an injected activity of 1480–2250 MBq and a 2D PET camera. Cancer risk projection models were based on the National Research Council Biological Effects of Ionizing Radiation VII report assuming a linear relationship with radiation exposure. In these conditions and considering the 9.1 million tests performed each year in the USA, the number of additional future cancers would be about 7400. This number should be viewed with great caution because, from the time of Berrington’s publication, estimated effective doses have been substantially decreased with the use of most advanced instrumentation allowing to dramatically decrease injected activities.

Considering the lower injected activity, which is now possible with 3D SPECT and PET cameras, which results in low effective doses, the number of additional and supposed cancers should be significantly lowered. Finally, the risk/benefit ratio should be taken into consideration for each individual patient before performing a myocardial perfusion test and the lowest activity should be injected compatible with a good image quality leading to an accurate diagnosis.

## Limitations

### Cost

Currently, the cost for one test with rubidium-82 is higher than with a ^99m^Tc-labeled radiopharmaceutical even in the favorable situation of a nuclear cardiology center with a high recruitment rate (Table 3). In the USA, sestamibi-^99m^Tc is currently reimbursed at about $70 per dose, usually using 2 doses. However, in the hospital setting, the entire procedure including the drug is paid at $1139. Rubidium-82 is reimbursed at approximately $250 per dose. In the hospital setting, the entire procedure is reimbursed $1286.

This relative low cost of technetium-99m is partly due to the fact that Mo-99/Tc-99m is not paid at full cost which explains some of the problems encountered nowadays with aging reactors and the difficulties to build new ones. Consequently, the use of rubidium-82 should be, at least at short term, limited to patients who are unable to complete exercise stress test, who are obese or who had a previous equivocal and non-conclusive SPECT test. Moreover, rubidium-82 should be used, despite its cost, when MBF and coronary flow reserve quantitation are required, for example, in patients with multi-vessel coronary disease.

Some new high-energy cyclotrons will be installed shortly and will enable to extend strontium-82 availability, and then provide generators in a cost effective model.

### Limited number and availability of PET 3D systems

To be as cost-effective as possible with a ^82^Sr/^82^Rb generator, it is necessary to test a high number of patients before its expiration date (presently 28–42 days) but this situation needs to have available a dedicated cardiac PET/CT camera. In Europe, where a ^82^Sr/^82^Rb generator has not been approved yet, there is no such dedicated PET/CT cameras and the conventional PET/CT cameras are mainly used in oncology with fluorodeoxyglucose (FDG). A minimum of 8–10 patients are tested each day with this radiopharmaceutical and up to a maximum of 15–20 patients. Consequently, a quite limited number of patients could be injected with rubidium-82 per day making each test relatively expensive. This situation reinforces the fact that, at the beginning, Rb-82 will be probably limited to selective cases (see Cost). It will take probably a few years before some dedicated cardiac PET/CT cameras are available in large cardiology centers with a recruitment rate warranting such equipment.

## Perspectives

Strontium-82, which is the parent nucleus of rubidium-82, is produced using proton beam interacting on a target containing stable rubidium. To be economically viable, an accelerator with proton beam of energy higher than 70 MeV and intensity >100 μA must be used. There are only few places in the world where such accelerators are available: Brookaven National Laboratory (BNL-USA), Los Alamos National Laboratory (LANL-USA), iThemba labs (South Africa), INR (Russia), Triumf (Canada), and Arronax (France). These accelerators are not dedicated facilities and only part of the beam time is devoted to this production. Recently, based on the success of the first prototype of a 70 MeV machine built by a commercial cyclotron provider and installed in Nantes (France), several private companies are considering the use of such commercial cyclotron for strontium-82 production. Zevacor (USA) is one of them and CDNM (Russia) is the other one. In parallel, a 70 MeV cyclotron is being installed at Legnaro (Italy) for research purpose but one of the two available beam lines may be used for radionuclide production. This indicates that the availability of strontium-82 in the future will be higher allowing to secure the supply chain and enlarge the use of this radionuclide to other countries (at the moment only north America is using it routinely), especially in Europe.

From a technological point of view, another advance will enlarge strontium-82 production, which is the use of rubidium metal target instead of the rubidium chloride target. With such change, higher yields can be obtained at the price of a more technical radiochemical process.

Finally, several designs of strontium-82/rubidium-82 generators are being studied and may reach the market in the future. This will allow to get generators with extended lifetime (up to 60 days) or lower activity which will better fit the need of users.

## Conclusion

There is no doubt that the advantages of ^82^Rb/PET myocardial imaging have been clearly documented since its routine clinical use after approval in the USA nearly 30 years ago. For a long time, its accuracy was significantly better than that obtained with the SPECT/^99m^Tc technology particularly in obese or overweight patients and women with large breasts due to attenuation correction artifacts. With the most recent hybrid SPECT imaging technology, the situation changed, leading to an improved specificity of ^99m^Tc/SPECT. Currently, the main advantage of ^82^Rb/PET is its capacity to accurately measure the MBF and flow reserve. Even if such measurement is technologically feasible with ^99m^Tc/SPECT, it will take some years before potential validation. Finally, given the very short physical half-life of rubidium-82, the radiation exposure rate with this radionuclide is significantly lower than with technetium-99m. Using 3D hybrid PET system, it is possible to inject low activities of rubidium-82 while maintaining a good image quality. The radiation exposure rate is then acceptable and inferior to natural exposure.

It can be anticipated that in the future ^82^Rb/PET and ^99m^Tc/SPECT will continue to be used according availability of hybrid cameras and radionuclides.

## Conflict of Interest Statement

The authors declare that the research was conducted in the absence of any commercial or financial relationships that could be construed as a potential conflict of interest.
